# EDTA Improves Stability of Whole Blood C-Peptide and Insulin to Over 24 Hours at Room Temperature

**DOI:** 10.1371/journal.pone.0042084

**Published:** 2012-07-30

**Authors:** Timothy J. McDonald, Mandy H. Perry, Roy W. A. Peake, Nicola J. Pullan, John O’Connor, Beverley M. Shields, Beatrice A. Knight, Andrew T. Hattersley

**Affiliations:** 1 Department of Clinical Chemistry, Royal Devon and Exeter NHS Foundation Trust, Exeter, United Kingdom; 2 NIHR Clinical Research Facility, Peninsula College of medicine and Dentistry, Barrack Road, University of Exeter, Exeter, United Kingdom; 3 Department of Clinical Biochemistry, NHS Grampian Health Board, Aberdeen, United Kingdom; 4 Clinical Pathology (City Campus), Nottingham University Hospitals NHS Trust, Hucknall Road, Nottingham, United Kingdom; Baylor College of Medicine, United States of America

## Abstract

**Introduction:**

C-peptide and insulin measurements in blood provide useful information regarding endogenous insulin secretion. Conflicting evidence on sample stability and handling procedures continue to limit the widespread clinical use of these tests. We assessed the factors that altered the stability of insulin and C-peptide in blood.

**Methods:**

We investigated the impact of preservative type, time to centrifugation, storage conditions and duration of storage on the stability of C-peptide and insulin on three different analytical platforms.

**Results:**

C-peptide was stable for at least 24 hours at room temperature in both centrifuged and whole blood collected in K^+^-EDTA and serum gel tubes, with the exception of whole blood serum gel, which decreased to 78% of baseline at 24 hours, (p = 0.008). Insulin was stable at room temperature for 24 hours in both centrifuged and whole blood collected in K^+^-EDTA tubes. In contrast insulin levels decreased in serum gel tubes both centrifuged and whole blood (66% of baseline, p = 0.01 and 76% of baseline p = 0.01, by 24 hours respectively). C-peptide and insulin remained stable after 6 freeze-thaw cycles.

**Conclusions:**

The stability of C-peptide and insulin in whole blood K^+^-EDTA tubes negates the need to conform to strict sample handling procedures for these assays, greatly increasing their clinical utility.

## Introduction

C-peptide is secreted together with insulin in equimolar quantities following the cleavage of insulin from pro-insulin in the pancreatic â-cells. The measurement of blood C-peptide and insulin has several clinical uses: The investigation of conditions resulting in excessive endogenous insulin secretion, as found in insulinoma [1] and in investigating the causes hypoglycaemic episodes (i.e. endogenous vs. exogenous source of insulin) [2]; the assessment of endogenous insulin secretion in people with diabetes to help define aetiology and best treatment [3], [4], [5]; and the assessment of insulin resistance [6].

Despite the established clinical application of blood C-peptide and insulin measurement, their widespread clinical use is limited by practical restrictions associated with sample collection: It is considered that both insulin and C-peptide concentrations rapidly decrease as a result of protease activity present in blood. At the time of writing, on the UK website “Assayfinder” (www.assayfinder.com) the majority of registered laboratories (10 out of 14) recommend collection of blood with ice followed by immediate centrifugation and frozen transportation to the laboratory [7].

Published evidence on stability of insulin in various anticoagulant and storage conditions is conflicting. Most studies suggest that insulin is stable on separated serum and plasma (EDTA and lithium heparin) when stored at both 4°C and −20°C for greater than five days [8], [9], [10]. However, insulin has been found to be less stable at room temperature in separated serum and plasma, with degradation of between 10% and 30% at 24 hours [8], [9], [10], [11]. Delayed separation of whole blood collected in EDTA plasma results in a dramatic decrease in activity to approximately 10% of baseline concentration after 24 hours and 70% after just 3 hours at room temperature [12]. In contrast, other studies suggest insulin is stable for between 4–5 hours in un-separated whole blood collected in both serum gel and plasma tubes at room temperature [13], [14]. The reason for the variation in reported stability is unclear, but it has been suggested that this could reflect differences between assays [11].

There is less information on the stability of C-peptide. C-peptide is stable for 24 hours on separated serum and plasma when stored at both at 4°C and room temperature [9]. In whole blood, C-peptide decreases to 44% of baseline concentration after four hours when collected into serum gel tubes at room temperature [14]. A separate study of whole blood collected into EDTA found C-peptide was stable for 24 hours at room temperature as well as 4°C. This suggests the possibility of increased stability in EDTA [11]. As with insulin, the variation in stability may be method specific as differences in stability of C-peptide at 2–8°C was found between chemiluminescence-based immunoassay method and a radio-immunoassay method [15].

The conflicting evidence of the stability of C-peptide and insulin and the strict recommended sample collection procedures for both C-peptide and insulin has restricted the routine use of these tests. The aim of this work was determine the impact of preservative type, time to centrifugation, storage temperature and duration of storage on the stability of C-peptide and insulin on three analytical platforms, with the aim of determining optimal pre-analytical sample handling procedures for these tests.

## Methods

### Subjects

5 healthy non-diabetic volunteers (3 male, 2 female), median age 29 years (range 24–35 years), BMI 24.2 kg/m^2^ (range 21.7–26.2) were recruited for the study. To achieve a range of blood insulin and C-peptide levels the volunteers ranged from fasting to post prandial status at blood sampling (C-peptide range 384−2658 pmol/L, insulin range 4.7–59.3 mU/L).

The study was approved by the Frenchay South West National Research Ethics Service Committee, UK. Written informed consent was obtained from all participants.

**Figure 1:**
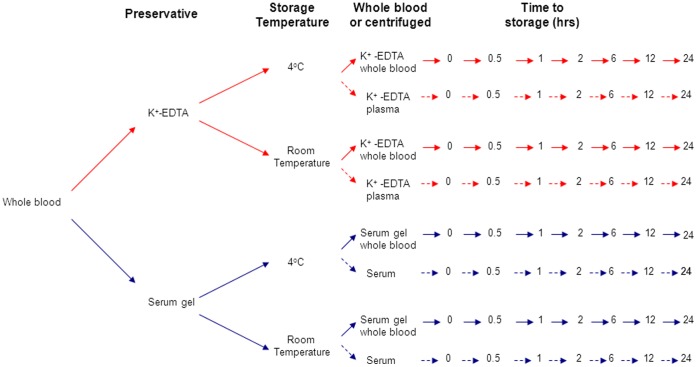
Flow diagram detailing the sample collection protocol for the preservative and stability study over 24 hours. At each time point the samples were centrifuged at 3000g for 10 minutes and the supernatant frozen at −80°C.

### Materials

C-peptide and insulin analyses were undertaken on three analytical platforms; Roche E170 analyser (Roche Diagnostics, Mannheim, Germany), Siemens ADVIA Centaur XP® and Siemens Immulite 2000 (Siemens Healthcare Diagnostics, Deerfield, IL, USA). Analysis on the E170 platform was undertaken at the Clinical Chemistry Department at the Royal Devon and Exeter NHS Foundation Trust, Exeter, UK. The C-peptide assay employs a direct electrochemiluminescence immunoassay utilising mouse monoclonal anti-C-peptide antibody labelled with ruthenium and a second mouse monoclonal anti-C-peptide antibody coupled to paramagnetic particles. The insulin assay uses the same technology employing two mouse monoclonal anti-insulin antibodies. Analysis on the Centaur was undertaken at the Department of Clinical Biochemistry, Aberdeen Royal Infirmary, NHS Grampian Heath Board, Scotland, UK. Both assays are two-site sandwich immunoassays using direct chemiluminescent technology. The insulin assay employs a mouse monoclonal anti-insulin antibody labelled with acridinium ester and a second mouse monoclonal anti-insulin antibody covalently coupled to paramagnetic particles. The C-peptide assay utilises the same technology with two mouse monoclonal anti-C-peptide antibodies.

**Figure 2:**
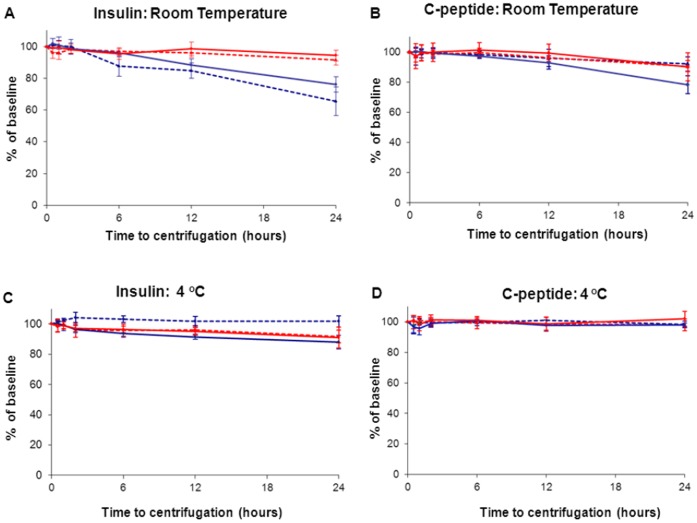
Stability of C-peptide and insulin in plasma and serum at room temperature and 4°C. Blood taken from 3 non-diabetic volunteers assayed on the E170, Centaur and Immulite 2000 platforms. The samples were stored at room temperature (top row; figures A and B) and in the fridge at 4°C (bottom row; figures C and D) at 0.5, 1.0, 2.0, 6.0, 12 and 24 hours before freezing at −80°C. Data presented as mean percentage of baseline at each time point, with error bars representing the 95% confidence intervals. Red lines indicates samples collected K^+^-EDTA, blue lines indicate blood collected into serum gel, solid lines indicate whole blood and dashed lines indicate sample centrifuge at baseline.

Analysis on the Immulite 2000 was undertaken at Clinical Pathology, Nottingham University Hospitals NHS Trust, Nottingham, UK. Both assays are sandwich immunoassays using direct chemiluminescence technology. The insulin assay uses a solid phase bead coupled to a mouse monoclonal anti-insulin antibody. The liquid phase consists of sheep polyclonal and mouse monoclonal anti-insulin antibodies, both covalently coupled to alkaline phosphatase. The C-peptide assay utilises the same technology but with mouse monoclonal anti-C-peptide antibodies. Siemens Healthcare Diagnostics do not recommend the use of potassium-EDTA (K^+^-EDTA) plasma for use with the Immulite 2000 C-peptide and insulin assays.

**Figure 3:**
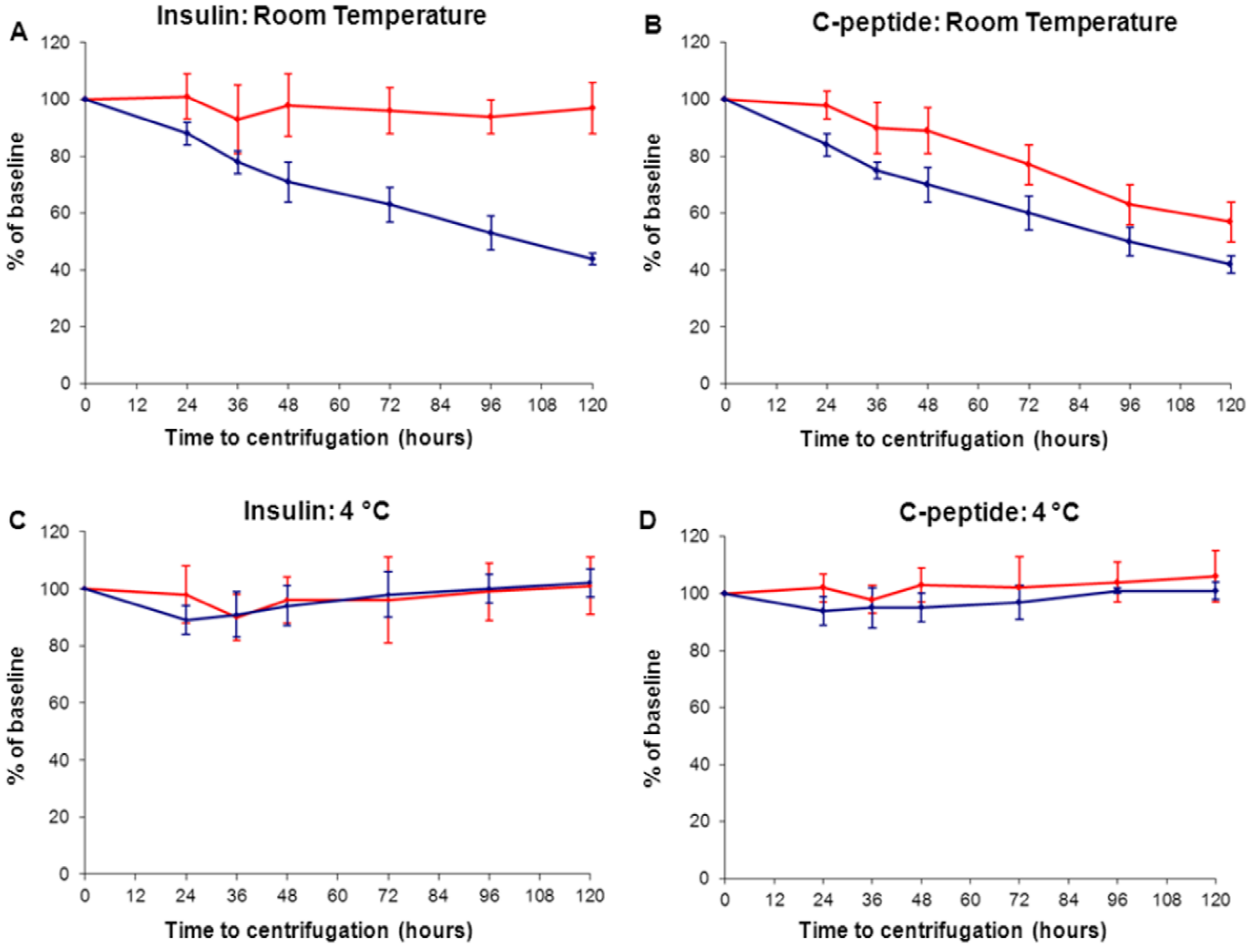
Five day stability of C-peptide and insulin in whole blood stored on K^+^-EDTA and serum gel at room temperature and 4°C. Blood taken from 5 non-diabetic volunteers assayed on the E170. The samples were stored at room temperature (top row; figures A and B) and in the fridge at 4°C (bottom row; figures C and D) at 0, 12, 24, 36, 48, 72, 96 and 120 hours before freezing at −80°C. Data presented as mean percentage of baseline at each time point, with error bars representing the 95% confidence intervals. Red lines indicate samples collected in K^+^-EDTA, blue lines indicate blood collected into serum gel.

All three C-peptide assays are standardised against WHO IS 84/510 [16] and all insulin assays against the WHO 1st IRP 66/304 reference standard preparations.

### 1. Stability of C-peptide and Insulin (Plasma and Serum)

#### 1.1 Preservative and stability study

A single blood (80 mL) sample was taken from 3 of the non-diabetic healthy volunteers. The blood was separated into 56 tubes: 28 collected into 2.7 mL, K^+^-EDTA containers (Sarstedt, Beaumont Leys, UK) and 28 collected into 2.7 mL serum gel containers (Sarstedt, Beaumont Leys, UK). Half of each tube type were stored at room temperature (average temperature 19.5°C) and the remaining half in the refrigerator at 4°C for 0, 0.5, 1, 2, 6, 12 and 24 hours. Half the samples for each tube type and temperature (n = 14 serum gel and n = 14 K^+^-EDTA) were centrifuged immediately to separate the serum/plasma from cells and the other half were left as whole blood (See figure 1). At each time point one of each sample type was centrifuged (if required) at 3000g for 10 minutes and the serum/plasma split into three aliquots to allow parallel analysis on the three platforms. Samples were stored frozen at −80°C until analysis was preformed as a batch.

C-peptide and insulin results are presented as mean percentage change from baseline ±95% confidence interval (CI). Percentage changes of insulin and C-peptide >10% from baseline concentrations were considered clinically significant. Differences between the baseline and the level of C-peptide and insulin at 24 hours were assessed by the Wilcoxon test.

#### 1.2 Extended stability study/delayed delivery of whole blood to the laboratory study

Blood samples were collected from all 5 of our non-diabetic healthy volunteers and the samples separated into 28 aliquots: 14 were collected into Sarstedt 2.7 mL, K^+^-EDTA containers (Sarstedt, Beaumont Leys, UK) and 14 collected into 2.7 mL Sarstedt serum gel containers. The tubes were either stored at room temperature (average temperature 19.5°C) or in the refrigerator at 4°C for 0, 024, 36, 48, 72, 96 and 120 hours. At each time point one of each sample type was centrifuged at 3000g for 10 minutes. Samples were stored frozen at −80°C until analysis was performed as a batch on the E170 platform.

C-peptide and insulin results are presented as mean percentage change from baseline ±95% confidence interval (CI). Percentage changes of insulin and C-peptide >10% from baseline concentrations were considered clinically significant. Differences between the baseline and the level of C-peptide and insulin at 24 hours were assessed by the Wilcoxon test.

#### 1.3 Freeze thaw study

Five separate blood samples were collected into K^+^-EDTA and serum gel tubes and stored at −80°C. Each sample was exposed to 6 freeze-thaw cycles in one week to assess the impact on C-peptide and insulin concentration. After the final thaw all the samples were mixed thoroughly before analysis on the E170 platform as a batch.

### 2. Analytical Characteristics of C-peptide Assay

#### 2.1 Assay bias and imprecision

Imprecision of the C-peptide and insulin assays was assessed using blood samples from healthy volunteers (n = 3) with 8 replicates. Data presented as mean ±95% CI. In the absence of a reference method, percentage bias was calculated against the all method mean for the three platforms.

**Table 1:** Freeze-thaw data. Data on the Roche platform.

Analyte	Sample Type	Number of freeze-thaw cycles					
		1	2	3	4	5	6
Insulin	Whole blood inserum gel	100 (±0)	106 (±9)	106 (±7)	105 (±14)	100 (±8)	99 (±7)
	Whole blood in EDTA	100 (0)	98 (±17)	107 (±9)	103 (±14)	105 (±7)	100 (±4)
C-peptide	Whole blood inserum gel	100 (0)	102 (±8)	103 (±5)	98 (±12)	98 (±7)	105 (±3)
	Whole blood in EDTA	100 (0)	99 (±4)	106 (±6)	104 (±10)	100 ±1)	101 (±3)

Expressed as percentage mean (±2 standard errors). Average % change from baseline (n = 5).

## Results

### 1. Stability of C-peptide and Insulin

#### 1.1 Storage and preservatives

The combined results of the 24 hours stability study for all three analysers are shown in Figure 2. Table S1 and S2 show the data obtained for the individual platforms.

Insulin levels did not drop below 90% of baseline over 24 hours at room temperature, for samples stored both as centrifuged and whole blood K^+^-EDTA (94% of baseline, p = 0.02 and 92%, p = 0.01 of baseline) (Figure 2A). In contrast, insulin concentration decreased in samples stored in serum gel tubes both centrifuged and whole blood (66% of baseline, p = 0.01 and 76% of baseline p = 0.01, at 24 hours respectively).

At 4°C, insulin levels in whole blood collected in K^+^-EDTA measured 91% of baseline (p = 0.01) and whole blood serum gel tubes dropped to 88% of baseline, (p = 0.01) (Figure 2C).

C-peptide concentration did not decrease below 90% of baseline in any sample stored at room temperature with the exception of whole blood serum gel samples, which decreased to 78% of baseline, (p = 0.008).

At 4°C, C-peptide remained stable in both whole blood collected in K^+^-EDTA and serum gel tubes for 24 hours (Figure 2D).

#### 1.2 Extended stability/delayed delivery to laboratory study

To investigate the effect of a delayed delivery of a whole blood sample to the laboratory, an additional 5 samples were investigated for stability under different storage conditions up to 120 hours after collection. Samples were analysed on the Roche E170 platform only.

The results of the extended 120 hours stability study for the E170 analyser is shown in Figure 3. Both Insulin and C-peptide were stable in whole blood in both K+-EDTA and serum gel for 120 hours when stored at 4°C (mean 102% of baseline). Insulin remains stable for 120 hours at room temperature on whole blood K^+^-EDTA plasma samples (mean 97% of baseline) and C-peptide for 36 hours on K^+^-EDTA plasma (mean 90% of baseline, p = 0.01) (Figure 3).

#### 1.3 Freezer Storage

There was no decrease in plasma and serum concentrations of either C-peptide or insulin after 6 freeze-thaw cycles (see table 1).

### 2. Analytical Characteristics of C-peptide and Insulin Assay

The analytical characteristics of the three platforms are summarised in Text S1 and Table S3.

## Discussion

Our study has identified that C-peptide and insulin are stable in unseparated whole blood collected in K^+^-EDTA for at least 24 hours at ambient room temperature. This finding negates the need for complex sample handling, facilitating more widespread clinical use of these tests.

The stability of both C-peptide and insulin in K^+^-EDTA preserved blood increases the clinical usefulness of these tests. We found that C-peptide is more stable, at room temperature, in whole blood K^+^-EDTA samples than serum gel tubes on all three platforms. This was also the case for insulin on the Centaur and E170 platforms. However, the manufacturer’s state that K^+^-EDTA plasma is not a suitable sample type for use with the Immulite 2000 insulin assay and as a result the optimal sample handling conditions on this platform is whole blood serum gel, which was stable for 6 hours (97% of baseline) at ambient room temperature. In the extended stability study we found no degradation in insulin levels for 5 days and C-peptide levels dropped less than 10% over 36 hours when stored in whole blood K^+^-EDTA, when analysed on the E170 platform. These results are in contrast to many reports which indicate that both C-peptide and insulin are unstable at room temperature on whole blood [8], [9], [12], [14]. In addition, we found that 6 freeze-thaw cycles over 5 days did not affect the levels of C-peptide or insulin in plasma or serum when analysed on the E170 platform. The implications of this data is that samples for insulin or C-peptide analysis can be sent to the laboratory from outpatient clinics and community doctors without any special storage conditions.

We found considerable differences in the analytical performance of the three platforms for both insulin and C-peptide. The data collected in our study highlights the well documented problem of poor assay agreement between the major immunoassay methods used in the UK to measure insulin and C-peptide [17], [18], [19]. Working groups have been set up to address the widely disparate values between analytical methods for both C-peptide and insulin to improve standardisation [17], [19], [20], [21]. The groups plan to combine efforts to improve standardisation with the aim of establishing a complete reference measurement system and certified primary reference materials based on pure biosynthetic insulin and C-peptide [20].

Our study has limitations: Insulin and C-peptide analysis was performed on only three analytical platforms and this could be expanded to other commercially available immunoassays. The extended stability analysis and freeze-thaw study was only performed on the Roche platform and the results may not be applicable to other assay platforms. All samples were stored at −80°C until analysis was performed as a batch. From the experiments performed in this study we cannot be sure that storage at −80°C storage does not alter C-peptide and insulin invitro. Finally, the effect of haemolysis on the stability of insulin and C-peptide was not assessed in this study. This would be an interesting question for future research.

In conclusion, our study has shown that insulin and C-peptide is stable in K+-EDTA blood for at least 24 hours at ambient room temperature. The stability at room temperature greatly increases the potential clinical utility of these tests.

## Supporting Information

Table S1 **Stability of C-peptide for each individual analyser, expressed as mean percentage of baseline (n = 3).**
(DOC)

Table S2 **Stability of insulin for each individual analyser, expressed as mean percentage of baseline (n = 3).**
(DOC)

Table S3 **Comparison of Roche, Centaur and Immulite 2000 platforms: Mean, Standard Deviation (SD), Coefficient of Variation (CV) calculated on n = 8 repeats for three levels of insulin and C-peptide.** * =  below detection limit of assay. b =  replicates n = 4
(DOC)

Text S1 **Imprecision and Methodological Bias of C-peptide and Insulin.**
(DOC)
