# Supplementary material for: EDTA Improves Stability of Whole Blood C-Peptide and Insulin to Over 24 Hours at Room Temperature
Source: PLoS One. 2012 Jul 30;7(7):e42084. doi: 10.1371/journal.pone.0042084 (PMC3408407; doi:10.1371/journal.pone.0042084)
Supplement: Table S1 — Stability of C-peptide for each individual analyser, expressed as mean percentage of baseline (n = 3). (DOC) [file pone.0042084.s001.doc]

|  |  | **C-peptide** | | | | | | | |
| --- | --- | --- | --- | --- | --- | --- | --- | --- | --- |
| **Analyser** | **Time before centrifugation (hours)** | **Centrifuged 4 ^o^C serum** | **Centrifuged 4^o^C plasma** | **Centrifuged Room temperature serum** | **Centrifuged room temperature plasma** | **Whole blood 4^o^C serum** | **Whole blood 4^o^C plasma** | **Whole blood room temperature serum** | **Whole blood room temperature plasma** |
| Centaur | 0 | 100 | 100 | 100 | 100 | 100 | 100 | 100 | 100 |
|  | 0.5 | 91 | 98 | 89 | 98 | 92 | 101 | 98 | 92 |
|  | 1 | 101 | 103 | 97 | 107 | 90 | 98 | 101 | 99 |
|  | 2 | 99 | 102 | 99 | 101 | 100 | 101 | 97 | 103 |
|  | 6 | 100 | 102 | 101 | 103 | 103 | 100 | 97 | 109 |
|  | 12 | 101 | 99 | 93 | 98 | 99 | 99 | 97 | 102 |
|  | 24 | 97 | 99 | 89 | 92 | 98 | 100 | 79 | 88 |
| Roche | 0 | 100 | 100 | 100 | 100 | 100 | 100 | 100 | 100 |
|  | 0.5 | 101 | 100 | 101 | 99 | 100 | 101 | 100 | 100 |
|  | 1 | 100 | 101 | 101 | 99 | 97 | 99 | 99 | 100 |
|  | 2 | 101 | 98 | 99 | 99 | 99 | 102 | 101 | 102 |
|  | 6 | 102 | 99 | 98 | 100 | 100 | 100 | 98 | 98 |
|  | 12 | 101 | 101 | 97 | 98 | 98 | 100 | 94 | 98 |
|  | 24 | 98 | 99 | 95 | 92 | 99 | 103 | 79 | 91 |
| Immulite | 0 | 100 | 100 | 100 | 100 | 100 | 100 | 100 | 100 |
|  | 0.5 | 101 | 102 | 100 | 94 | 97 | 101 | 101 | 100 |
|  | 1 | 99 | 100 | 99 | 93 | 100 | 99 | 101 | 99 |
|  | 2 | 97 | 101 | 101 | 95 | 99 | 101 | 100 | 95 |
|  | 6 | 97 | 98 | 96 | 95 | 99 | 102 | 97 | 97 |
|  | 12 | 101 | 97 | 98 | 92 | 97 | 97 | 88 | 98 |
|  | 24 | 100 | 96 | 93 | 87 | 97 | 103 | 76 | 91 |

**Supporting Information Table S2-** Stability of C-peptide for each individual analyser, expressed as mean percentage of baseline (n = 3)
