# Supplementary material for: EDTA Improves Stability of Whole Blood C-Peptide and Insulin to Over 24 Hours at Room Temperature
Source: PLoS One. 2012 Jul 30;7(7):e42084. doi: 10.1371/journal.pone.0042084 (PMC3408407; doi:10.1371/journal.pone.0042084)
Supplement: Table S2 — Stability of insulin for each individual analyser, expressed as mean percentage of baseline (n = 3). (DOC) [file pone.0042084.s002.doc]

|  |  | **Insulin** | | | | | | | |
| --- | --- | --- | --- | --- | --- | --- | --- | --- | --- |
| **Analyser** | **Time before centrifugation (hours)** | **Centrifuged 4^o^C serum** | **Centrifuged 4^o^C plasma** | **Centrifuged Room temperature serum** | **Centrifuged room temperature plasma** | **Whole blood 4^o^C serum** | **Whole blood 4^o^C plasma** | **Whole blood room temperature serum** | **Whole blood room temperature plasma** |
| Centaur | 0 | 100 | 100 | 100 | 100 | 100 | 100 | 100 | 100 |
|  | 0.5 | 95 | 103 | 102 | 97 | 104 | 97 | 102 | 97 |
|  | 1 | 97 | 103 | 99 | 97 | 99 | 98 | 102 | 99 |
|  | 2 | 95 | 105 | 104 | 101 | 99 | 95 | 98 | 99 |
|  | 6 | 96 | 103 | 86 | 97 | 96 | 97 | 97 | 94 |
|  | 12 | 96 | 101 | 88 | 97 | 92 | 95 | 90 | 99 |
|  | 24 | 92 | 102 | 67 | 89 | 93 | 86 | 79 | 92 |
| Roche | 0 | 100 | 100 | 100 | 100 | 100 | 100 | 100 | 100 |
|  | 0.5 | 103 | 100 | 102 | 95 | 99 | 102 | 102 | 101 |
|  | 1 | 103 | 102 | 100 | 95 | 99 | 100 | 101 | 99 |
|  | 2 | 102 | 103 | 96 | 96 | 97 | 100 | 101 | 97 |
|  | 6 | 100 | 103 | 93 | 97 | 93 | 96 | 99 | 97 |
|  | 12 | 99 | 102 | 87 | 94 | 92 | 95 | 92 | 98 |
|  | 24 | 97 | 101 | 66 | 94 | 92 | 96 | 81 | 96 |
| Immulite | 0 | 100 | * | 100 | * | 100 | * | 100 | * |
|  | 0.5 | 97 | * | 98 | * | 98 | * | 101 | * |
|  | 1 | 99 | * | 103 | * | 100 | * | 100 | * |
|  | 2 | 91 | * | 99 | * | 94 | * | 97 | * |
|  | 6 | 92 | * | 84 | * | 92 | * | 92 | * |
|  | 12 | 93 | * | 79 | * | 91 | * | 83 | * |
|  | 24 | 86 | * | 63 | * | 79 | * | 69 | * |

**Supporting Information Table S3-** Stability of insulin for each individual analyser, expressed as mean percentage of baseline (n = 3)
