# Supplementary material for: EDTA Improves Stability of Whole Blood C-Peptide and Insulin to Over 24 Hours at Room Temperature
Source: PLoS One. 2012 Jul 30;7(7):e42084. doi: 10.1371/journal.pone.0042084 (PMC3408407; doi:10.1371/journal.pone.0042084)
Supplement: Table S3 — Comparison of Roche, Centaur and Immulite 2000 platforms: Mean, Standard Deviation (SD), Coefficient of Variation (CV) calculated on n = 8 repeats for three levels of insulin and C-peptide. * = below detection limit of assay. b = replicates n = 4 (DOC) [file pone.0042084.s003.doc]

| **C-peptide (pmol/L)** | | | | | | | | | | | | | |
| --- | --- | --- | --- | --- | --- | --- | --- | --- | --- | --- | --- | --- | --- |
| **sample** | **Roche** | | | | **Centaur** | | | | **Immulite 2000** | | | | **All method mean** |
|  | **mean** | **SD** | **CV** | **% bias** | **mean** | **SD** | **CV** | **% bias** | **mean** | **SD** | **CV** | **% bias** |  |
| Low | 384 | 13.9 | 3.6 | 33.6 | 230 | 22.2 | 9.6 | -19.8 | 248 | 27.9 | 11.3 | -13.8 | 287 |
| Medium | 1789 | 65.6 | 3.7 | 18.4 | 1216 | 32.7 | 2.7 | -19.5 | 1527 | 64.8 | 4.2 | 1.1 | 1511 |
| High | 2658 | 55.0 | 2.1 | 10.2 | 2107 | 215.6 | 10.2 | -12.6 | 2471 | 276.9 | 11.2 | 2.4 | 2412 |
| **Mean** |  |  | **3.1** | **20.7** |  |  | **7.5** | **-17.3** |  |  | **8.9** | **-3.4** |  |
| **Insulin (mU/L)** | | | | | | | | | | | | | |
| **sample** | **Roche** | | | | **Centaur** | | | | **Immulite 2000** | | | | **All method mean** |
|  | **mean** | **SD** | **CV** | **% bias** | **mean** | **SD** | **CV** | **% bias** | **mean** | **SD** | **CV** | **% bias** |  |
| Low | 4.7 | 0.8 | 16.5 | -3.7 | 5.1 | 0.8 | 15.3 | 3.7 | * | * | * | * | 4.9 |
| Medium | 36.9 | 2.2 | 5.9 | -2.6 | 43.4 | 2.0 | 4.6 | 14.6 | 28.7^b^ | 1.0 | 3.4 | -24.1 | 37.9 |
| High | 59.3 | 2.3 | 3.8 | -5.0 | 72.2 | 7.1 | 9.8 | 15.7 | 51.43^b^ | 1.12 | 2.18 | -17.6 | 62.4 |
| **Mean** |  |  | **8.7** | **-3.8** |  |  | **9.9** | **11.4** |  |  | **2.8]** | **-20.8** |  |

**Supporting Information Table S4 Comparison of Roche, Centaur and Immulite 2000 platforms:** Mean, Standard Deviation (SD), Coefficient of Variation (CV) calculated on n=8 repeats for three levels of insulin and C-peptide. * = below detection limit of assay. ^b^= replicates n=4
