# Supplementary material for: EDTA Improves Stability of Whole Blood C-Peptide and Insulin to Over 24 Hours at Room Temperature
Source: PLoS One. 2012 Jul 30;7(7):e42084. doi: 10.1371/journal.pone.0042084 (PMC3408407; doi:10.1371/journal.pone.0042084)
Supplement: Text S1 — Imprecision and Methodological Bias of C-peptide and Insulin. (DOC) [file pone.0042084.s004.doc]

***Supporting Information Text S1***

*Imprecision: C-peptide*

Intra-assay imprecision (mean %CV) ranged from 3.1% (E170 method) to 8.9% (Immulite method).

*Methodological Bias: C-peptide*

There was considerable variation in C-peptide concentration between the three platforms. In the absence of reference methodology, bias was calculated against the all-method mean for the three platforms. The maximum C-peptide concentrations were found on the E170 platform, with a mean positive bias of 20.7% and the minimum observed with the Centaur assay demonstrating a negative bias -17.3% (Supporting Information Table S4).

*Imprecision: Insulin*

Mean Intra assay CV was lowest in the Immulite 2000 assay (2.8%) and the highest CV was in the Centaur assay (9.9%). However the low level sample in the Immulite 2000 was below the limit of detection for the assay, quoted by the manufacturer as being 2.0 mU/mL (Supporting Information Table S4).

*Methodological Bias: Insulin*

There was less variation in the insulin assays than the C-peptide but there was still considerable variation in the measured insulin levels between the three platforms. In the absence of reference methodology, bias was calculated against the all-method mean for the three platforms. The maximum insulin concentrations were found on the Centaur, with a mean positive bias of 11.4%. Minimum concentrations were observed in the Immulite 2000 assay with a negative bias of -20.8% (Supporting Information Table S4).

**Supporting Information Text S1**- Imprecision and Methodological Bias of C-peptide and Insulin
